# Prostatectomy in Two Stages

**Published:** 1900-11-03

**Authors:** 


### PROSTATECTOMY IN TWO STAGES.

Among the interesting papers read at Ipswich was one by Mr. Bruce Clarke in which he described the method he has adopted for removing the prostate in two stages, first performing a supra-pubic cystotomy and thoroughly cleansing the bladder; and then, when the urine has become "sweet, clear, and acid," placing the patient in the lithotomy position, introducing a grooved sound, making an incision in the perineum and shelling out the prostate. In the seven cases in which this plan of treatment was decided on it was carried out with complete success in five. In one the second part of the operation was not proceeded with because complete relief was obtained by the first stage. In the remaining case the second half was abandoned because the patient's condition did not seem to warrant further operative proceedings. When this patient was last heard of, however, he was fairly comfortable, his supra-bupic wound having healed How far this operation is to be regarded as generally applicable in the ordinary run of cases is a question into which Mr. Bruce Clarke does not enter. There are other operations, such as castration and vasectomy, which have had good success in many cases, but in hard fibrous prostates these proceedings are not likely to be of any avail, and there will probably always be a residuum in which it may be found necessary to remove the gland rather than wait in the hope of it shrivelling. In such cases there is much to be said on general surgical principles in favour of the double operation, a procedure by which several great advantages are gained; first, that the bladder can be cleansed and some approach to an aseptic condition can be obtained before the prostate is touched; second, that the surgeon during the operation has the benefit of the finger within the bladder to guide and assist the one by which the actual enucleation is performed; and third, that the drainage afterwards is far better than in any case of purely supra-pubic operation. If increased experience should show continued success this operation may be found applicable not merely to the residuum but to many of those cases for which at present vasectomy appears to offer the most hopeful prospect of relief.

